# The toxic effects of alizarin red S on catalase at the molecular level†

**DOI:** 10.1039/c9ra02986a

**Published:** 2019-10-17

**Authors:** Shimeng Hu, Dong Yuan, Yang Liu, Lining Zhao, Hongli Guo, Qigui Niu, Wansong Zong, Rutao Liu

**Affiliations:** School of Environmental Science and Engineering, Shandong University, America CRC for Environment & Health, Shandong Province 72# Jimo Binhai Road Qingdao Shandong 266237 P. R. China rutaoliu@sdu.edu.cn +86 531 88365489 +86 531 88365489; Department of Chemistry and Chemical Engineering, Qilu Normal University 36# Lishan Road Jinan 250013 P. R. China; College of Population, Resources and Environment, Shandong Normal University 88# East Wenhua Road Jinan 250014 P. R. China

## Abstract

Alizarin red S (ARS) is a widespread mordant dye derived from alizarin. However, it was reported to be mutagenic and carcinogenic probably because it could induce oxidative damages in organisms. Catalase (CAT) is an important antioxidant enzyme defensing oxidative damages induced by xenobiotics. The underlying mechanisms of ARS interacting with CAT have not been clarified yet. This study is conducted to characterize the functional and conformational changes on CAT by ARS and the binding details to further investigate their interaction mechanisms. Under exposure of ARS at 5 μM, CAT activity was significantly decreased to 76.2%. Inhibition of CAT probably resulted in promotion of intracellular oxidative stress and pro-oxidant property of ARS. The interaction between ARS and CAT was proved to be spontaneous and exothermic. However, limited structural changes were observed according to spectroscopic results. Results showed that ARS prefers to bind with residues buried in the active site and could alter the activity of CAT, which were agree with the molecular docking results. This work proves the adverse effects of ARS on CAT mainly at molecular level and further highlights its potential risks to heath.

## Abstract 2

Alizarin red S is confirmed to be toxic to catalase at molecular level attributing to the structural and functional changes of catalase.

## Graphical abstract

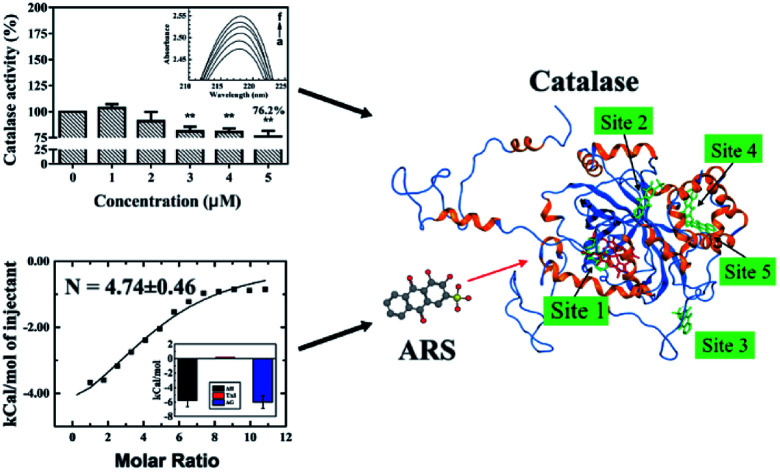

## 1. Introduction

Catalase (CAT) is a metallo enzyme with heme groups embedded in its polypeptide chains and generally considered as the primary defence against oxidative damage induced by xenobiotics.^1^ CAT could catalyze the disproportionation progress of hydrogen peroxide into water and oxygen. Hydrogen peroxide is the by-product of mitochondrial electron transportation, β-oxidation of fatty acids and so on, which is considered as an important factor causing oxidative stress in organisms.^2,3^

Alizarin and its derivatives (such as alizarin red S, ARS) have been widely used as a series of mordant dyes in textile industries due to their vivid and durable properties.^4^ Comparing with other alizarin derivatives, ARS is more water soluble and multifunctional, such as the application in histological studies to identifying calcium in tissues and vital staining of bones.^5–7^ According to the structure shown in Fig. S1,† ARS is a typical anthraquinone dye consisting of two neighboring hydroxyl groups.^8^ ARS shares similar structure with flavonoids, another major class of polyphenols from plants and fungi that has been extensively studied.^9^ Recent investigations indicate that flavonoids are inhibitors of many enzymes including CAT, resulting in Reactive Oxidative Species (ROS) increment and subsequently leading to cell death in cancerous cells.^10,11^ Flavonoids are therefore considering beneficial rather than detrimental when it comes to induction of apoptosis of cancer cells.^12^ Conformational changes of CAT by flavonoids and the characterization of their binding details has also been investigated in recent studies.^9,11^

Comparing to flavonoids, researches on interactions between ARS and biomolecules in organisms have not been conducted in depth. Recent studies has demonstrated that ARS could induced structural and functional changes to serum albumins.^13,14^ ARS was also reported to introduce adverse effects to organisms, such as oxidative damages.^4,15^ Many diseases including inflammation and tumor are related to the disorder of redox balance. The maintenance of redox level is closely in connection with antioxidant enzymes including CAT.^16–19^ However, interactions between ARS and antioxidant enzymes including CAT are still unclarified yet. Investigation on the interactions between exogenous substances and enzymes *in vitro* could reflect their adverse effects and binding mechanisms directly.^20,21^ In this work, multiple spectroscopic methods, calorimetric measurements and molecular docking simulations were utilized to investigate the structure and function changes of CAT under ARS exposure. Further intracellular ROS measurements on mouse primary hepatocytes in absence or presence of ARS exposure were also conducted, in order to investigate the possible oxidative stress changes in consequence of CAT activity changes. Thermodynamic parameters and binding modes of CAT–ARS system were also analyzed to recognize the binding details. This study intended to characterize the interaction between ARS and CAT at molecular level, and better understand the potential adverse effects of ARS.

## 2. Experimental section

### 2.1. Materials

ARS and CAT from bovine liver were purchased from Beijing Solarbio Ltd. Na_2_HPO_4_·12H_2_O and NaH_2_PO_4_·2H_2_O were purchased from Tianjin Damao Chemical Reagent Factory. Phosphate buffered solution (PBS, pH 7.2–7.4) for intracellular oxidative stress analysis was from Beijing Macgene Ltd., Beijing, China. Dulbecco's modified Eagle's medium (DMEM), fetal bovine serum, and penicillin/streptomycin were all purchased from Thermo Fisher Scientific, Waltham, MA, USA. Dimethyl sulfoxide (DMSO) was provided by from Biofroxx (Germany). Hydrogen peroxide was bought from Sinopharm Chemical Reagent Co., Ltd. Phosphate buffer (a mixture of Na_2_HPO_4_·12H_2_O and NaH_2_PO_4_·2H_2_O solution, pH 7.4) was chosen to control the solution pH. All the water solutions involved in this work were prepared by using ultrapure water, and stock solutions were preserved at 0–4 °C.

### 2.2. Apparatus

All the absorbance measurements were carried out on SHIMADZU UV-2450 spectrophotometer (Shimadzu, Japan). Fluorescence measurements were performed on HITACHI FL-4600 fluorescence spectrophotometer (Hitachi, Japan). Circular dichroism measurements were measured on J-810 CD spectrometer (Jasco, Japan). Calorimetric experiments were carried out on MicroCal ITC200 isothermal titration microcalorimeter with a workstation. ROS measurements were evaluated on a FACS AriaIII flow cytometry (BD, US).

### 2.3. UV-visible absorption measurements

Samples were prepared as followings: different concentrations of ARS, 1 mL fixed concentration of CAT solution and 1 mL phosphate buffers (0.2 M) were added in centrifuge tubes and diluted to 10 mL by using ultrapure water. The samples were then placed at 298 K for 30 min to control the condition of interaction. The UV-visible spectra were recorded in a wavelength range of 190–450 nm. There were no sample where the concentration of ARS was greater than 10 μM. According to UV-Vis spectrum of 10 μM ARS (the maximum concentration) shown in Fig. S2,† the absorbance of ARS at around 218 nm, 280 nm, and 406 nm (peaks in CAT spectra) was weak and near the valleys in UV-Vis spectrum of ARS. To further rule out the absorption of ARS, additional samples without CAT were prepared and used as references. The final spectra of each sample have removed the corresponding absorbance of ARS and buffer automatically by the SHIMADZU UV-2450 spectrophotometer.

### 2.4. Fluorescence measurements

Samples for the fluorescence measurements were prepared by adding increasing amount of ARS into CAT, similarly to UV-Vis absorption measurements. A sample of CAT solution with no ARS addition was chosen as the control group. Structural information could be learned by comparing fluorescence spectra of CAT and ARS-contaminated CAT.

Fluorescence spectra were collected on HITACHI FL-4600 fluorescence spectrophotometer at a scanning speed of 1200 nm min^−1^. Emission wavelength ranged from 290 nm to 450 nm. Excitation wavelength was set to 278 nm and photo-multiplier tube (PMT) voltage was limited to 600 V.

Synchronous fluorescence spectra measurements were set as Δ*λ* = 15 nm and Δ*λ* = 60 nm, respectively. Excitation wavelength ranged from 250 nm to 330 nm and PMT voltage was limited to 600 V.

Resonance light scattering (RLS) measurements were measured by setting Δ*λ* to 0, scanning from 230 nm to 500 nm.

For the three-dimensional fluorescence spectra, emission wavelength was set from 220 nm to 500 nm, while the excitation wavelength ranged from 200 nm to 400 nm.

### 2.5. Enzyme activity assay

CAT activity is reflected by its decomposition ability of hydrogen peroxide which has a strong absorbance at 240 nm.^22^ The inhibition rate of CAT activity was calculated by the following equation:^23^1
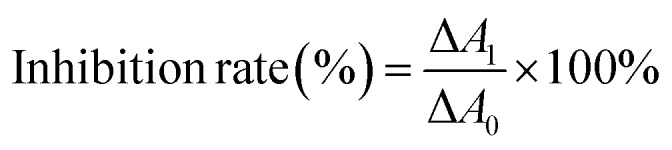
where, Δ*A*_1_ and Δ*A*_0_ are the absorbance reduction at 240 nm after the same period of time, since mixing up hydrogen peroxide with CAT contaminated by ARS and pure CAT sample, respectively.

Samples were prepared as the UV-Vis absorption measurements described. Absorption of hydrogen peroxide at 240 nm were measured every 30 seconds after mixing up diluted hydrogen peroxide with the samples. A sample without ARS was chosen as the control group to calculate the inhibition rates of CAT by ARS.

Additionally, measurements on positive and negative controls were also performed. The absorption values at 240 nm were measured every half minute after mixing up positive or negative control sample with hydrogen peroxide. The changes of absorption at 240 nm along time for positive and negative controls were shown in Fig. S3.† Positive control was a denatured CAT sample that proved the decomposition ability of CAT for hydrogen peroxide. It also proved that hydrogen peroxide would not get decomposed by itself in such a short time during absorption measurements. Negative control was a ARS solution without CAT addition, which was designed to verify that ARS cannot decompose hydrogen peroxide.

### 2.6. Circular dichroism (CD) measurements

CD spectra were recorded on circular dichroism spectrometer using a 0.1 cm quartz cell in a range from 190 nm to 260 nm at 298 K with the scanning speed set as 200 nm min^−1^. Sample preparation in CD measurements was similar to UV-Vis absorption measurements. Secondary structural changes of CAT by ARS can be informed by comparing to the control group where no ARS was added. An additional blank sample of phosphate buffer solution was prepared to correcting the baseline. Each CD spectrum was obtained by scanning for three times to record the average data. The baseline was automatically removed by the J-810 CD spectrometer. Results of CD spectra were analyzed by utilizing the CD Pro software (http://lamar.colostate.edu/∼sreeram/CDPro/), which helps to reckon the secondary structure contents of CAT.^24^

### 2.7. Isothermal titration calorimetry (ITC) measurements

Thermodynamic parameters of this system were performed on MicroCal ITC200 at 298 K. During the ITC measurements, all the solutions for the titration process were prepared using 0.02 M buffer to remove possible heat changes induced by mixing up two different solvents. To rule out the interference of ARS dilution process, an additional titration that injected ARS simply into phosphate buffer was performed to obtain the baseline.

Before titration start, CAT (20 μM) and ARS (1 mM) were loaded into sample cell and the syringe (40 μL in total), respectively. Total injection was set up to 14 times. First injection was set to 0.4 μL while each of the following 13 injections at an interval of 120 seconds was set to 3.0 μL per drop. Stirring speed was set at 1000 rpm. Curve-fittings for the titration data were analyzed by using Origin 7.0 software (Microcal LLC). The baseline obtained from blank titration was removed before curve-fitting using the same software.

### 2.8. Molecular docking study

To further characterize the binding details between CAT and ARS, a molecular docking study was performed by utilizing the Molecular Operation Environment (MOE, Version 2014, Chemical Computing Group Inc, Canada). The crystal structure of CAT from bovine liver (PDB code: 1TGU) was obtained from RCSB Protein Data Bank (http://www.rcsb.org/). In the preprocessing steps, essential protonation and partial charges were performed. The parameters were set as follows: Placement: Triangle Matcher; Rescoring 1: London dG, Refinement: Forcefield.

### 2.9. Measurement of intracellular ROS in primary hepatocytes

The complete medium was prepared using DMEM, fetal bovine serum, and penicillin/streptomycin in a ratio of 89 : 10 : 1. Adult male C57BL/6 J mice of 6 weeks were purchased from Shandong University Laboratory Animal Centre (Jinan, China). Mouse primary hepatocytes from liver tissue was obtained as described by Seglen *et al.*^25^ The isolated hepatocytes were centrifuged at 1500 rpm for 10 min at 4 °C and then washed using cold PBS for three times.^26^ ARS solution used in intracellular oxidative analysis was dissolved in the complete medium. The isolated hepatocytes were planted in 96-well plates with complete medium and exposed to different concentrations of ARS for 24 h under 5% CO_2_ at 37 °C. The blank sample was just isolated hepatocytes in complete medium without ARS exposure. To measure intracellular ROS, the fluorescent probe CM-H_2_DCFDA was utilized. It was non-fluorescent before that the highly fluorescent 2′,7′-dichlorofluorescein (DCF) was converted from it by the intracellular ROS. The 10 μM application solution of the probe was dissolved using 9 μL DMSO and then diluted by 9 mL PBS. The hepatocytes after incubation were centrifuged and washed by PBS twice times, and then incubated in dark with CM-H_2_DCFDA probe at 37 °C for 1 h. After washing by PBS again, the hepatocytes were suspended in 100 μL PBS, and evaluated on a FACS AriaIII flow cytometry (BD, US).

## 3. Results and discussion

### 3.1. CAT activity changes induced by ARS

**Fig. 1:**
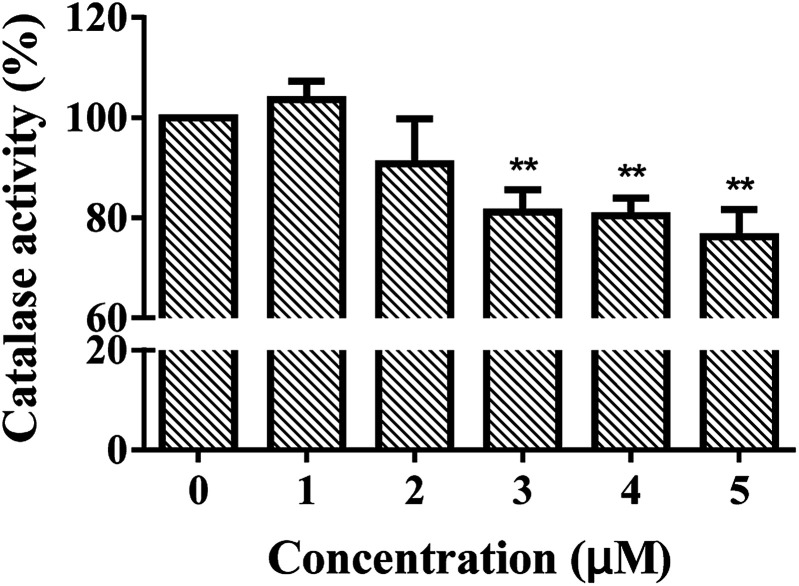
Effect of ARS on the activity changes of CAT. Conditions: CAT: 1 μM; ARS/(μM): 0, 1, 2, 3, 4, 5; pH 7.4; *T* = 298 K; *A*_H_2_O_2__ ≈ 0.55 (*p < 0.05, **p < 0.01, ***p < 0.001 compared with the control).

The structures of enzymes are directly relative to their behavior and functionality. CAT is a tetramer with four identical subunits, each of which consists of 506 residues.^22^ As shown in Fig. 1, CAT activity decreased as a whole along with ARS addition. A slight promotion (merely 103.6%, *p*-value is greater than 0.05) was observed when the concentration of ARS was 1 μM. With gradually increasing ARS concentrations, the CAT activity was inhibited obviously (the inhibitory rate is up to 23.8% at a concentration of 5 μM, *p*-value is less than 0.01). According to the results shown in Fig. S3,† ARS was proved to have no influences on hydrogen peroxide, hydrogen peroxide would not get decomposed by itself in such a short time of measurement, and CAT did catalyze the decomposition of hydrogen peroxide. Therefore, it could be concluded that CAT activity was generally inhibited by direct exposure of ARS at molecular level. This result kept consistent with the inhibition on CAT by flavonoids, which shared similar structures with ARS.^10^

As a biomarker, the inhibition of CAT could reflect the redox state in organisms and the effects of pollutants on organisms. To determine the possible production of oxidative stress introduced by the inhibition of CAT, the intracellular ROS levels in absence and presence of ARS were measured. Structural alternation is a possible reason for the activity changes on enzymes. We further studied the molecular mechanism of CAT with ARS multiple spectroscopic, calorimetric and molecular docking methods.

### 3.2. The intracellular oxidative stress induced by ARS

**Fig. 2:**
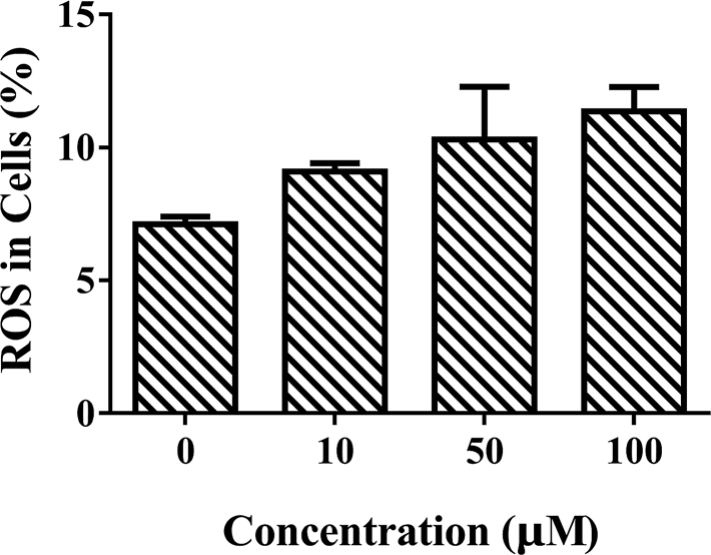
Effects of ARS exposure to ROS of mice primary hepatocytes. Conditions: hepatocyte: 10^7^ cells per mL; ARS (μM): (a) 0, (b) 10, (c) 50, (d) 100.

Liver plays an important role in metabolism and oxidative processes, and is the main organ of hydrocarbon detoxification and CAT distribution.^27,28^ Therefore, isolated primary hepatocytes was chosen to evaluate the effects of ARS. Mouse primary hepatocytes were treated with various concentrations of ARS for 24 h to evaluate the intracellular ROS levels. The intracellular ROS levels in absence and presence of various concentrations of ARS were shown in Fig. 2. Compared with the blank control, percentage of the hepatocytes with ROS increased from 7.1% to 12.4% when the concentration of ARS went up to 10^−4^ M. The slight promotion to intracellular ROS levels by ARS was in accordance with the inhibition of CAT activity, considering that inhibition of antioxidant enzymes such as CAT could lead to production of oxidative stress.^16–19^ Studies on flavonoids, another major class of polyphenolic compounds that shared similar structure with ARS, also suggested that inhibition of CAT by flavonoids resulted in promotion of ROS.^11^ Therefore, inhibition of CAT by ARS would probably result in the increased intracellular oxidative stress level of hepatocytes and pro-oxidant property of ARS.

### 3.3. Structural and conformational changes of CAT induced by ARS

#### 3.3.1. The changes of polypeptide backbone structure in CAT by ARS

**Fig. 3:**
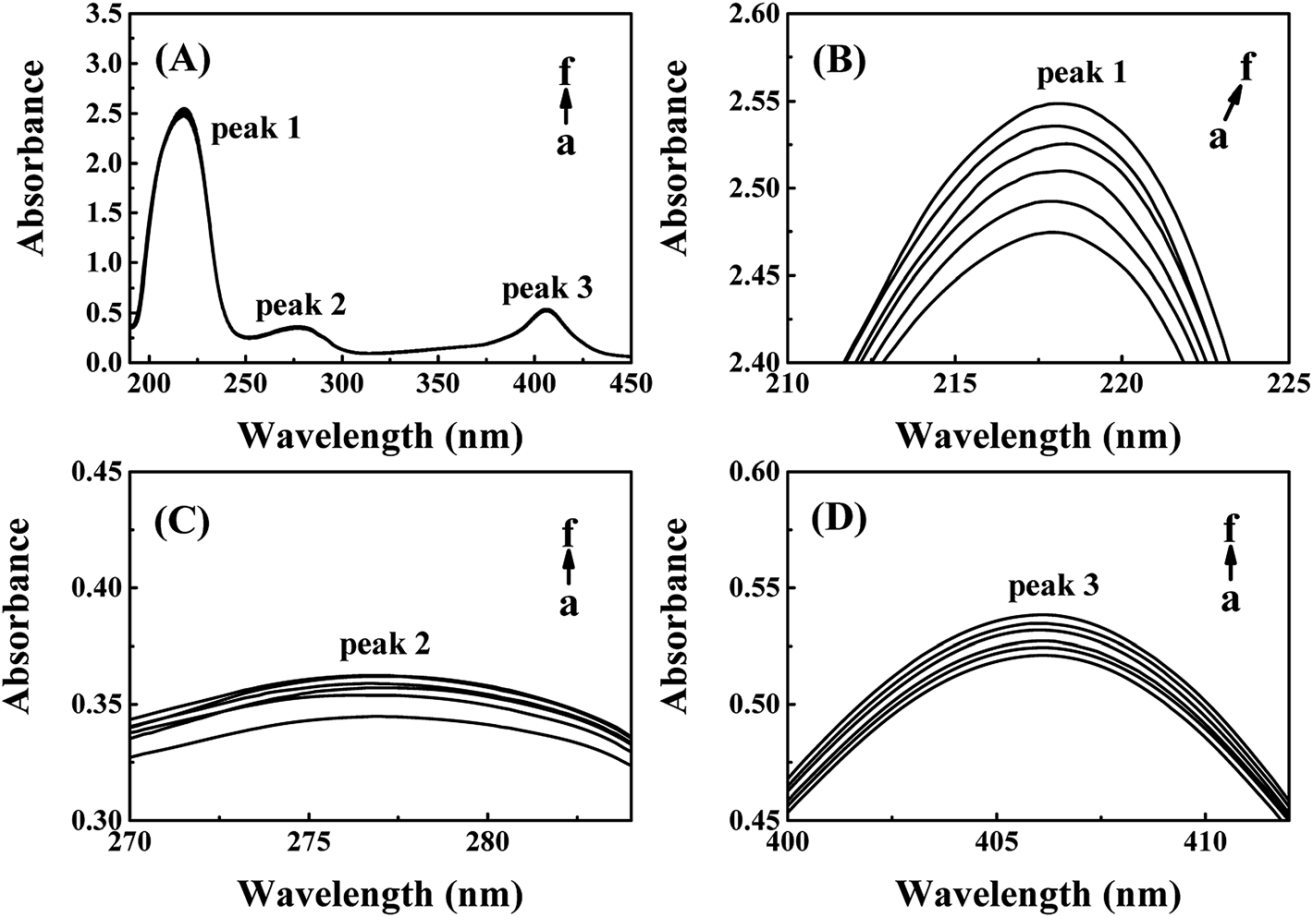
(A) UV-Vis spectra of CAT–ARS system; (B) peak of peptide backbone; (C) peak of aromatic amino acids residues; (D) peak of the heme groups. Conditions: CAT: 1 μM; ARS/(μM): (a–f) 0, 2, 4, 6, 8, 10; pH 7.4; *T* = 298 K.

UV-Vis spectroscopy measurements were designed to explore the backbone structure changes of CAT with or without exposure to ARS. Specific absorption peaks could reflect the detailed structural changes of enzymes. The UV-Vis absorption spectra of CAT with different concentrations of ARS is shown in Fig. 3(A). Three partial enlarged plots of absorption peaks were also represented in the Fig. 3.

The absorption peak 1 at around 218 nm is the characteristic peak of peptide backbone, which reflects the information of CAT framework.^28–30^ As shown in Fig. 3(B), peak 1 increased slightly with a red shift from 217.8 nm to 218.8 nm while the concentrations of ARS increased. The framework conformation of CAT is therefore slightly changed by ARS.^28,31,32^ The peak 2 at 280 nm is the characteristic peak of aromatic amino acids residues, which reflects the micro-environment on Trp, Tyr and Phe residues.^20,28^ There was a slight increment of absorption while no significant peak-shift could be observed in the peak 2 from Fig. 3(C). Therefore, the micro-environment of aromatic amino acids became slightly less hydrophobic. The peak 3 at 406 nm is the porphyrin-Soret band, which reflects the information of heme groups in CAT active center.^33^ As indicated in Fig. 3(D), the peak 3 was slightly promoted with no obvious peak-shift. It is suspected that ARS could contact with the amino acid residues near the heme groups and consequently inhibited the activity of CAT.^28^ To summarize, ARS only has slight impact on the peptide backbone, aromatic amino acids residues and porphyrin in CAT.

#### 3.3.2. Effects of ARS on secondary structures of CAT

To further investigate the potential impact of ARS on the secondary structure of CAT, CD spectra measurements were carried out.^22^ The spectra of CAT with or without ARS were illustrated in Fig. S4.† There are two negative peaks at 208 and 218 nm reflecting α-helix and β-sheet structures respectively. As listed in Table 1, the secondary structures in CAT altered slightly when concentrations of ARS increased. Under ascending concentrations of ARS, α-helix fraction in CAT slightly decreased (about 1.9%), while β-sheet fluctuated without obvious tendency, indicating a partial unfolding of CAT.^34^ Reduction of α-helix was also reported to be observed on CAT contaminated by various kinds of flavonoids, which were similar to ARS in structure.^11^ In general, slight adverse effects was found on the secondary structures of CAT by ARS, in consistent with the results of UV-Vis spectra.^35^

**Table 1:** Effects of ARS on the fractions of secondary structural in CAT

Concentration of ARS (μM)	Secondary structural content in enzyme (%)			
	α-Helix	β-Sheet	β-Turn	Unordered
0	32.0	24.8	23.5	33.6
2	29.4	27.8	24.0	35.3
4	29.2	28.2	23.5	34.4
6	31.1	24.8	23.3	33.5
8	29.9	28.4	23.2	34.0
10	30.3	23.5	20.2	29.3
50	30.1	24.6	21.0	31.5

#### 3.3.3. Size changes of CAT by ARS

**Fig. 4:**
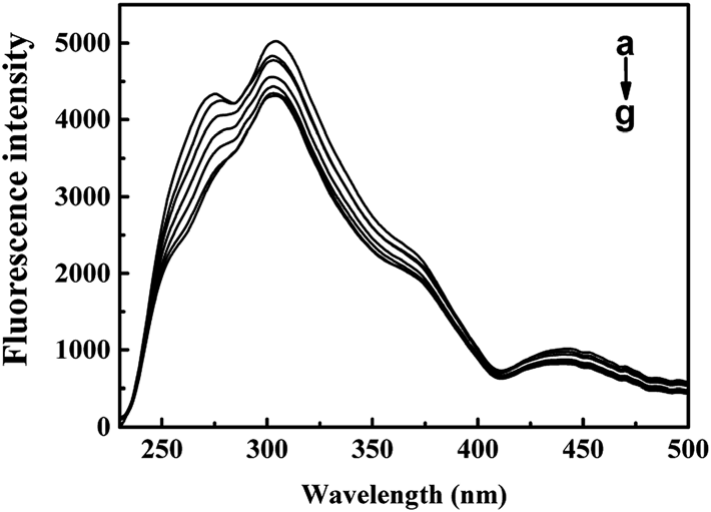
RLS spectra of CAT with different concentrations of ARS. Conditions: CAT: 1 μM; ARS/(μM): (a–g) 0, 1, 2, 3, 4, 5, 6; pH 7.4; *T* = 298 K.

RLS spectra is convenient to study the particle size changes. RLS intensity of the protein–ligand complex system is presented in proportion to its hydration radius.^36^ As shown in Fig. 4, RLS intensity of CAT–ARS system decreased slightly with the addition of ARS, indicating that the particle sizes of the system were reduced.^28^ ARS is likely to change the overall structure of CAT and therefore to reduce its hydration radius, which is probably caused by tightening of the four monomers in CAT.^35^

#### 3.3.4. Micro-environmental changes of amino residues in CAT

**Fig. 5:**
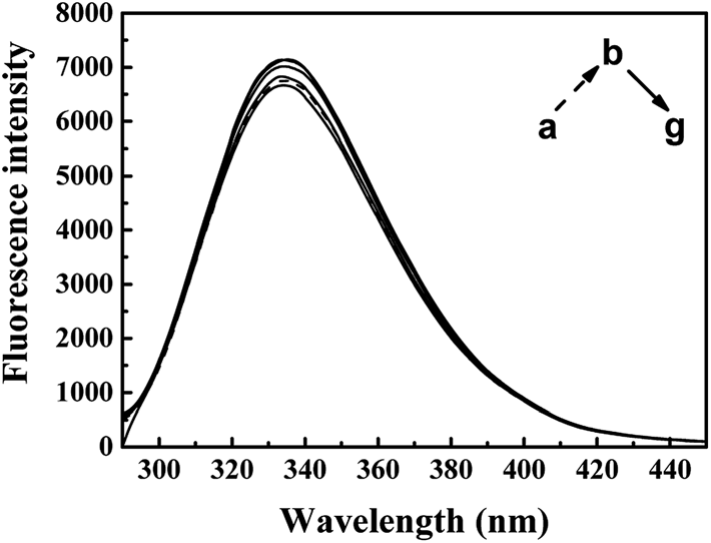
Effect of ARS on CAT fluorescence after IFE correction. Conditions: CAT: 1 × μM; ARS/(μM): (a–g) 0, 1, 2, 3, 4, 5, 6; pH 7.4; *T* = 298 K.

Fluorescence spectroscopy methods are rapid and effective to study the micro-environment of aromatic amino acid residues changes in proteins.^30^ Intrinsic fluorescence of protein is mainly emitted from its fluorophores, including Trp, Tyr and Phe residues.^37,38^ There are six Trp and twenty Tyr residues in a single monomer of CAT molecules from bovine liver. The micro-environment around these fluorophores could be investigated by analyzing fluorescence spectra and synchronous fluorescence spectra. The fluorescence spectra of CAT with or without exposure to ARS are shown in Fig. 5. Considering the inner-filter effect (IFE) introduced by the absorbance of the CAT–ARS system at the excitation and emission regions, possible interference of IFE could be corrected using the following equation:^39^2
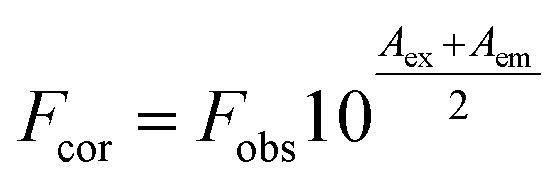
where, *F*_cor_ and *F*_obs_ are the corrected and observed fluorescence intensity respectively. *A*_ex_ and *A*_em_ are absorbance of the sample at excitation and maximum emission wavelengths.^40^

The inner-filter absorbance as illustrated in Fig. S5† indicates that the inner-filter effect could not be ignored.^13^ As shown in Fig. 5, the strong peak at about 334 nm firstly increased and then decreased. The peaks generally stagnated in a narrow range of fluorescence intensity with no obvious peak shift. It could be inferred from the figure that ARS interacted with the CAT molecule, but only has slight influences on the overall conformation changes.^41^ Micro-environment around fluorophores in CAT, including Trp and Tyr residues, was slightly affected by ARS.

**Fig. 6:**
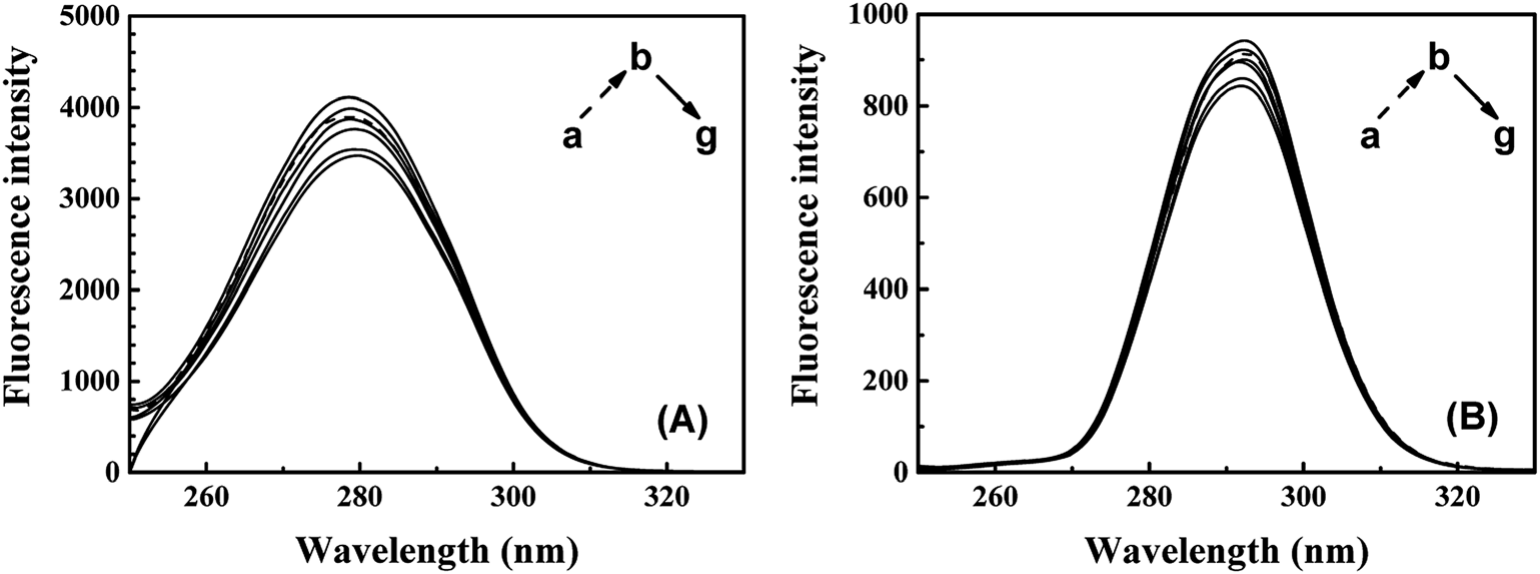
Synchronous fluorescence spectra of CAT–ARS system. Conditions: CAT: 1 μM; ARS/(μM): (a–g) 0, 1, 2, 3, 4, 5, 6; pH 7.4; *T* = 298 K; (A) Δ*λ* = 60 nm; (B) Δ*λ* = 15 nm.

The synchronous fluorescence of specific wavelength intervals (Δ*λ*) provides a quick and convenient approach to obtain the characteristic information for the micro-environment of specific residues, with less interferences and spectra overlapping.^42,43^ When the wavelength intervals (Δ*λ*) of synchronous fluorescence are fixed at 60 nm or 15 nm, the characteristic information for Trp or Tyr residues could be acquired respectively.^44^ As shown in Fig. 6, the fluorescence intensity of synchronous fluorescence spectra after IFE correction firstly increased and then kept decreasing. Slight red shift could be observed in the spectra for Trp residues, where the wavelength interval is 60 nm. Meanwhile, the spectrum for Tyr residues, where wavelength interval was fixed to 15 nm, has shown no obvious peak-shift. Similar red shift of the synchronous spectra for Trp residues in CAT exposed to flavonoids was also observed in recent studies.^9^ The red shift of synchronous spectra for Trp residues indicated that a less hydrophobic micro-environment around the Trp residues in CAT was caused by the exposure to ARS.^22,38^

**Fig. 7:**
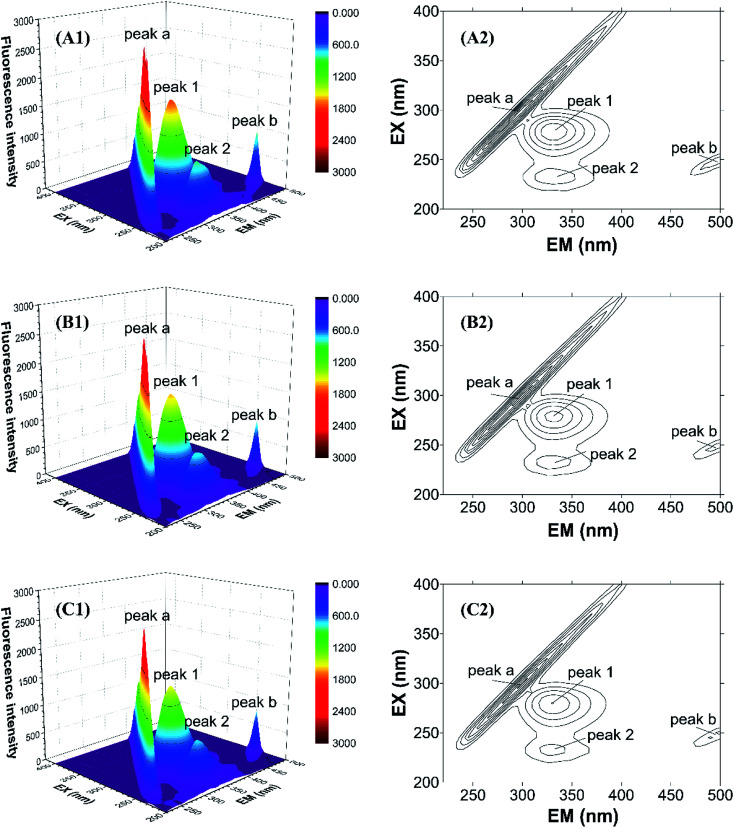
Three-dimensional fluorescence spectra of CAT–ARS system. Conditions: CAT: 1 μM; ARS/(μM): (A) 0; (B) 2; (C) 4; pH 7.4; *T* = 298 K.

To further study the micro-environmental changes in CAT, three-dimensional measurements were performed. As shown in Fig. 7, peak (a) and peak (b) are the Rayleigh scattering peak (*λ*_em_ = *λ*_ex_) and the secondary-order scattering peak (*λ*_em_ = 2*λ*_ex_), respectively.^32^ Both the two peaks decreased after the addition of ARS, indicating that the hydration radius decreased under the exposure of ARS. This result is corresponding to what could be learned from RLS spectra.

There are also another two fluorescence peaks marked as peak (1) and peak (2). Peak (1) reflects the characteristic of n → π* transition of amino acid residues and polypeptide backbone in CAT, while peak (2) is for the characteristic of π → π* transition of amino acid residues.^32^ The peak (1) mainly reflects the amino acid residues. It could be inferred from the figure that ARS slightly unfolded the backbone of ARS, in accordance with the quench of the two peaks.^32^ Micro-environment and conformation of CAT was changed, which confirmed the result of synchronous fluorescence spectra.

### 3.4. Thermodynamic characterization and binding affinity between ARS and CAT

ITC measurement was performed to characterize the binding affinity between ARS and CAT *via* investigating the thermodynamic parameters of this system, including binding constant (*K*), number of binding sites (*n*), enthalpy changes (Δ*H*), and entropy changes (Δ*S*). These thermodynamic parameters were then obtained directly by curve-fitting.^30,45^ The calorimetric profile carried with enthalpy changes, entropy changes and Gibbs free energy (Δ*G*) was shown in Table 2, in which the Gibbs free energy was calculated by the following equation:^46^

3 Δ*G* = Δ*H* − *T*Δ*S* = −*RT* ln *K*

where, *R*, *T* and *K* in the above mentioned equation is the gas constant, the temperature, and the binding constant, respectively.

**Table 2:** Thermodynamic parameters of the interaction between ARS and CAT

*N*	*K* (M^−1^)	Δ*H* (cal mol^−1^)	Δ*S* (cal mol^−1^ K^−1^)	Δ*G* (cal mol^−1^)
4.74 ± 0.46	(2.55 ± 0.75) × 10^4^	−5793 ± 853	0.727	−6009 ± 853

**Fig. 8:**
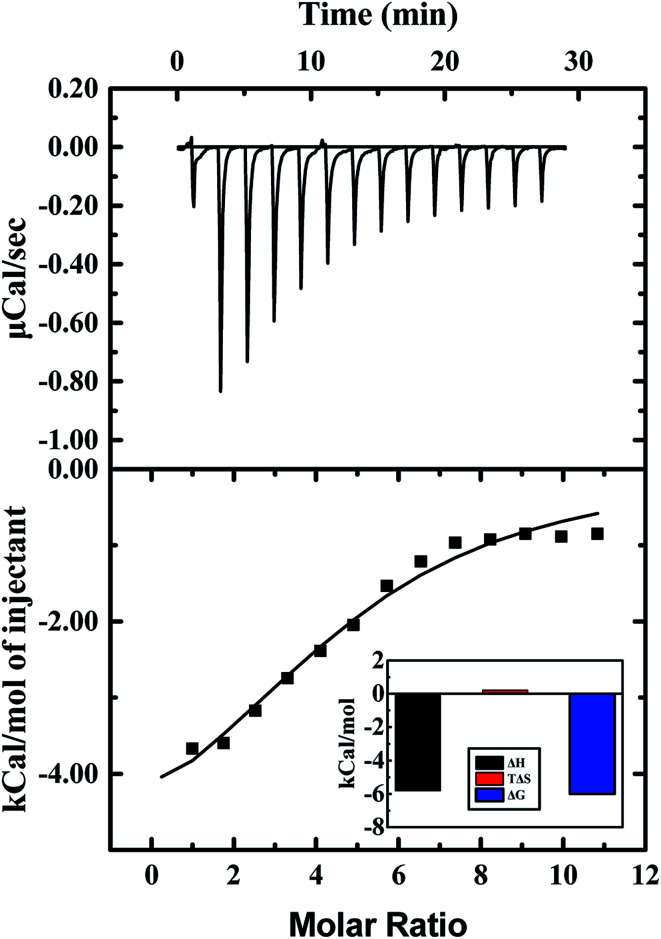
ITC results of interaction of CAT with ARS. Conditions: 298 K; CAT: 20 μM; ARS: 1 mM; pH 7.4; *T* = 298 K.

Heat changes during ITC measurements and the curve-fitting results are shown in Fig. 8. The top half shown that the heat flow fluctuating along titration, and the bottom half demonstrates the result of titration. Interaction between ARS and CAT was observed to be an exothermic process. The bottom half was analyzed by using the one binding site model, which was best fitted with the data retrieved from titration. The curve-fitting results indicated that the enthalpy changes (Δ*H*) of binding process were negative, while the entropy changes (Δ*S*) were positive-valued. Therefore, Gibbs free energy was calculated to be negative, which suggested that the binding process with (4.74 ± 0.46) binding sites between ARS and CAT was spontaneous. Furthermore, the predominant force of the interaction was proved to be electrostatic force according to its positive enthalpy changes and negative entropy changes.^47^

### 3.5. The binding model of ARS with CAT

**Fig. 9:**
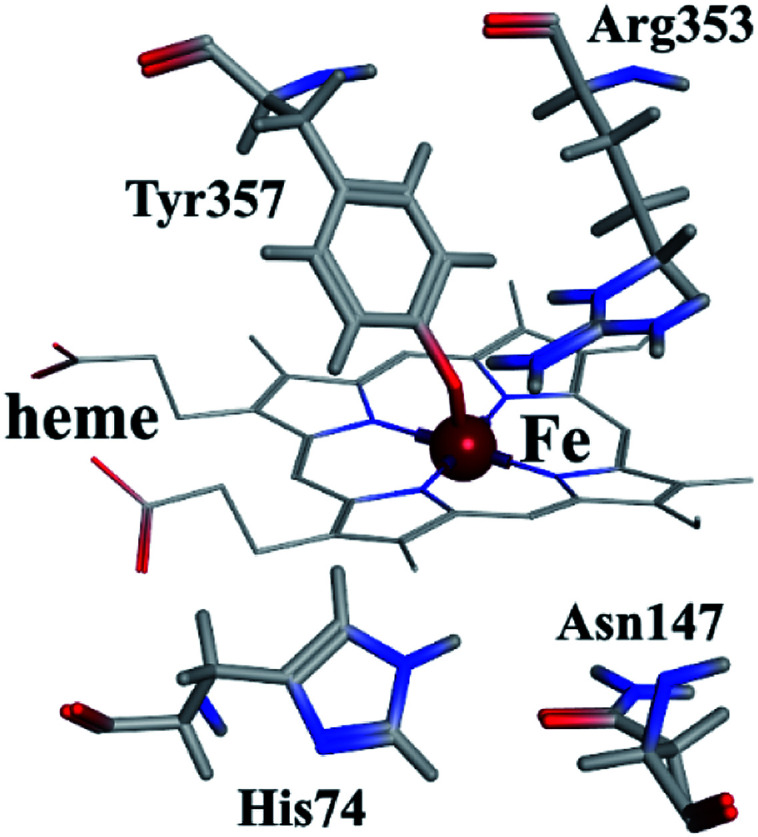
Heme group and its neighboring amino acid residues.

As shown in Fig. 9, the active site in each subunit of CAT is made up of one iron atom, one heme group and several amino acid residues neighboring to the heme group together.^48^ His 74 and Asn 147 residues enhance CAT catalyst efficiency by promoting chance for hydrogen peroxide molecules entering into the heme cavity.^48^ They interact with the nearby porphyrin ring in heme group *via* van der Waals force. Meanwhile, Tyr 357 (a ligand of the iron center) could activate the CAT active site and Arg 353 would influence Tyr 357 indirectly.^49^

To further investigate the possible binding sites of ARS on CAT molecule, the molecular docking simulation utilizing MOE was adopted in this study. In the docking simulations, a monomer of CAT was chosen as the target in order to better demonstrate the possible docking sites considering its relative large molecule.^28^

**Fig. 10:**
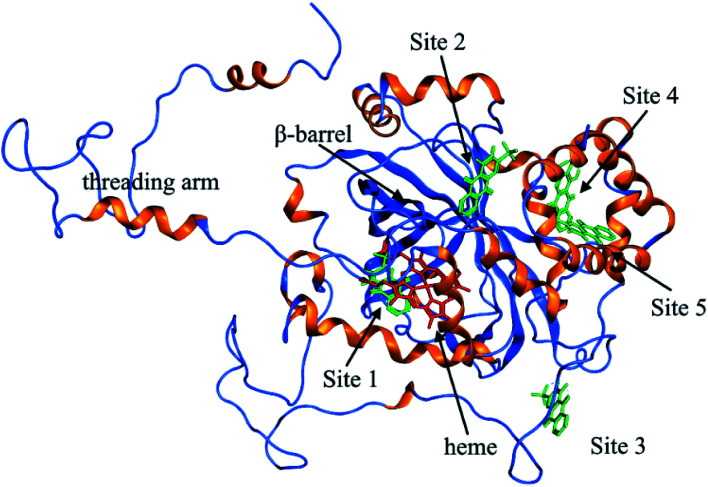
Binding interaction between the ligand ARS and the receptor CAT.

**Fig. 11:**
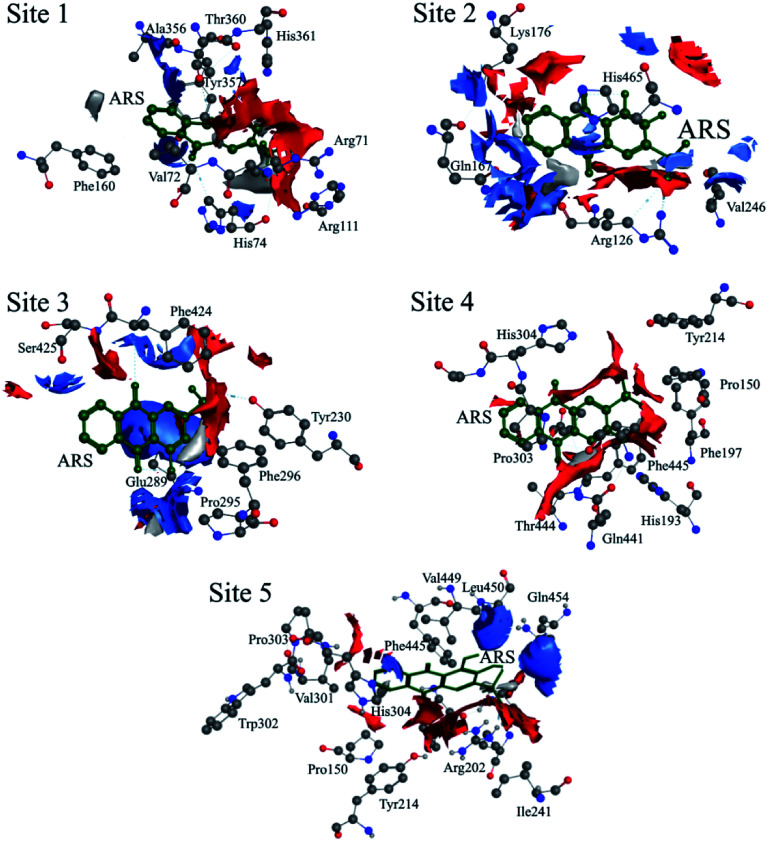
Electrostatic maps superimposed with ARS and CAT (positive charge region is highlighted in blue, negative in red and neutral in white).

According to the previous ITC experiment, there were (4.74 ± 0.46) binding sites on CAT, and the interaction between ARS and CAT was dominated by electrostatic force. Therefore, the top five binding sites with the lowest energy values calculated were chosen. These binding sites along with corresponding electrostatic maps were illustrated in Fig. 10 and 11 labelled from site 1 to site 5. The interaction between ARS and amino acid residues in CAT on these five binding sites was illustrated in Fig. S6,† which implied that ARS bound to CAT mainly by formation of H-bonds and ionic bonds. As shown in Fig. 11, electrostatic forces were the dominated driving force for these five results, in consistence with the previous ITC experiments.

ARS bound to these five probable binding sites from lower binding energy to higher by sequence. Site 1 of the lowest binding energy was located at the activity center of CAT neighboring to the porphyrin ring, while site 2 to 5 were located at the surface of CAT. According to Fig. S6 and Table S1,† ARS tended to form H-bond with the key residue His 74 in the activity center. Another key residue of CAT activity, Tyr 357, was also located in the binding pocket as illustrated in Fig. S6.† To further validate the binding, His 74 residue was mutated into Phe. The interactions between ARS and residues in site 1 before and after mutation were illustrated in Fig. S7† and corresponding binding parameters were listed in Table S1.† ARS would still bind to Arg 111 and Arg 71, but would not interact with the mutated residue anymore. This result confirmed the direct binding between ARS and His 74 *via* H-bond. It was suspected that the CAT activity changes by ARS were resulted from its influences on the key residues His 74 and Tyr 357. After site 1 is saturated, interactions between ARS and CAT at site 2 tended to hinder GLN 167 from forming hydrogen bonds with water molecule.^50^ Subsequently, diffusion process of hydrogen peroxide molecule to the active site was impeded by ARS, therefore indirectly decreased the activity of CAT.^49^ These results can explain the possible reason for the significant inhibition of CAT activity by ARS, while only little impact on the peptide backbone, aromatic amino acids residues and porphyrin in CAT observed according to UV-Vis spectra. Similar observations concerning that flavonoids could inhibit the activity of CAT partially due to the formation of H-bonds between flavonoids and CAT has been reported earlier.^10^ Meanwhile, in consistence with the former fluorescence and synchronous fluorescence spectra, ARS could also influence several aromatic amino acid residues such as Tyr 357 (site 1), Tyr 230 (site 3), and Tyr 214 (site 4, 5) and therefore changes the micro-environment of tyrosine residues.

## 4. Conclusions

This work reported the interactions between ARS and the antioxidant enzyme CAT to study the functional and structural changes of CAT under ARS exposure mainly at molecular level. ARS significantly inhibited the enzyme activity, and slightly changed the framework of CAT. Slightly conformational and structural changes of CAT have been observed in spectroscopic studies. Meanwhile, the contents of secondary structures in CAT were alternated. Measurements on intracellular oxidative stress level also suggested that the functional and structural changes on CAT by ARS would probably result in promotion of intracellular ROS level and pro-oxidant property of ARS. Thermodynamic characterization proved that the binding between ARS and CAT was both exothermic and spontaneous. There were 4.74 ± 0.46 binding sites. Further molecular docking simulation has found out the top five possible binding sites between CAT and ARS. The simulation results suggested that ARS would bind with the key residue His 74 *via* H-bond and probably influenced another key residue Tyr 357 in the binding pocket. This work has clarified the adverse effects of ARS on CAT mainly at molecular level and illustrates a strategy to evaluate the xenobiotics by investigating the structural and functional changes of biomacromolecules.

## Conflicts of interest

There are no conflicts to declare.

## Supplementary Material

RA-009-C9RA02986A-s001
