# Supplementary material for: The toxic effects of alizarin red S on catalase at the molecular level
Source: RSC Adv. 2019 Oct 17;9(57):33368–77. doi: 10.1039/c9ra02986a (PMC9073274; doi:10.1039/c9ra02986a)
Supplement: RA-009-C9RA02986A-s001 [file RA-009-C9RA02986A-s001.pdf]

Electronic Supplementary Material (ESI) for RSC Advances.
This journal is © The Royal Society of Chemistry 2019

Supplementary information

The toxic effects of Alizarin Red S to catalase at molecular level

*Shimeng Hu[a], Dong Yuan[b], Yang Liu[b], Lining Zhao[a], Hongli Guo[a], Qigui Niu[a], Wansong Zong[c], Rutao Liu[a,b]**

[a] School of Environmental Science and Engineering, Shandong University, America CRC for Environment & Health, Shandong Province, 72# Jimo Binhai Road, Qingdao, Shandong 266237, P.R. China

[b] Department of Chemistry and Chemical Engineering, Qilu Normal University, Jinan 250013, P.R. China

[c] College of Population, Resources and Environment, Shandong Normal University, 88# East Wenhua Road, Jinan 250014, P.R. China

*All correspondence should be addressed to:

Rutao Liu

School of Environmental Science and Engineering,

Shandong University, Qingdao, Shandong province, 72# Jimo Binhai Road, Qingdao, Shandong 266237, P.R. China

Phone/Fax: 86-531-88365489

Email: rutaoliu@sdu.edu.cn

P.R. China

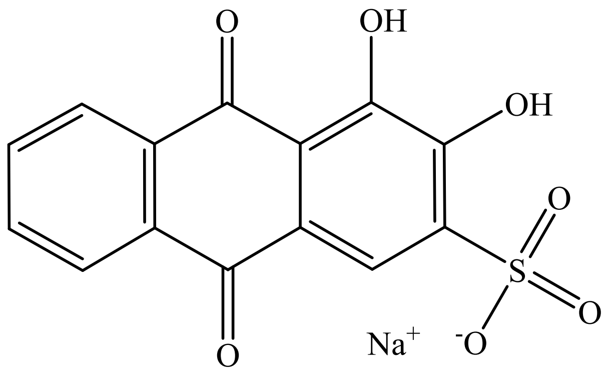

Fig. S1 Structure of Alizarin Red S.

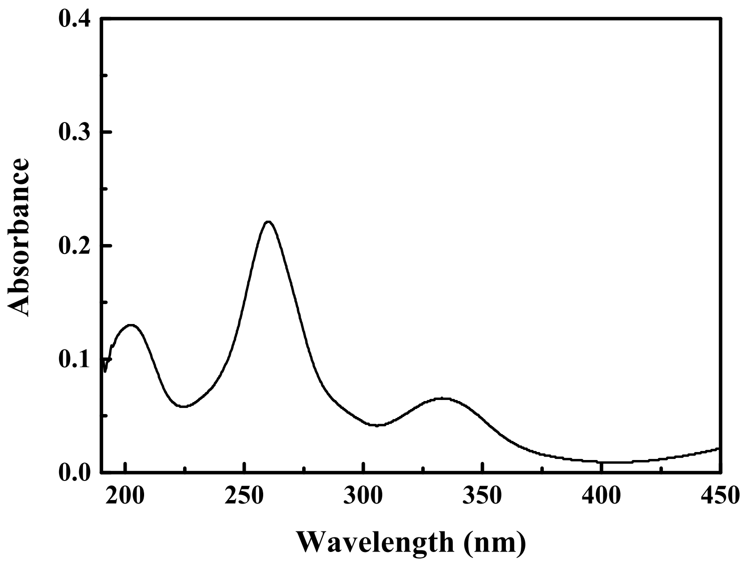

Fig. S2 UV-Vis spectrum of Alizarin Red S.

Conditions: ARS: 10 μM; pH 7.4; T = 298 K.

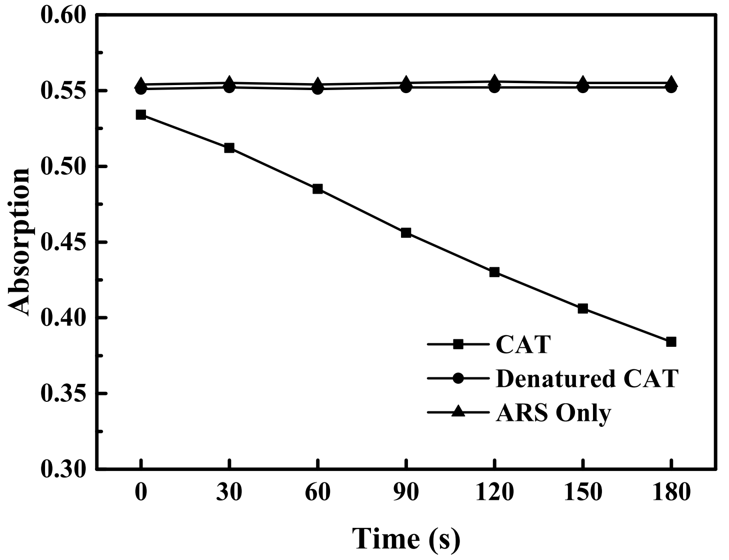

Fig. S3 Absorption change of hydrogen peroxide along with time.

Conditions: CAT: 1 μM CAT; Denatured CAT: 1 μM CAT that was denatured by heating and ultrasonic wave; ARS Only: 10 μM ARS; pH = 7.4; T = 298 K.

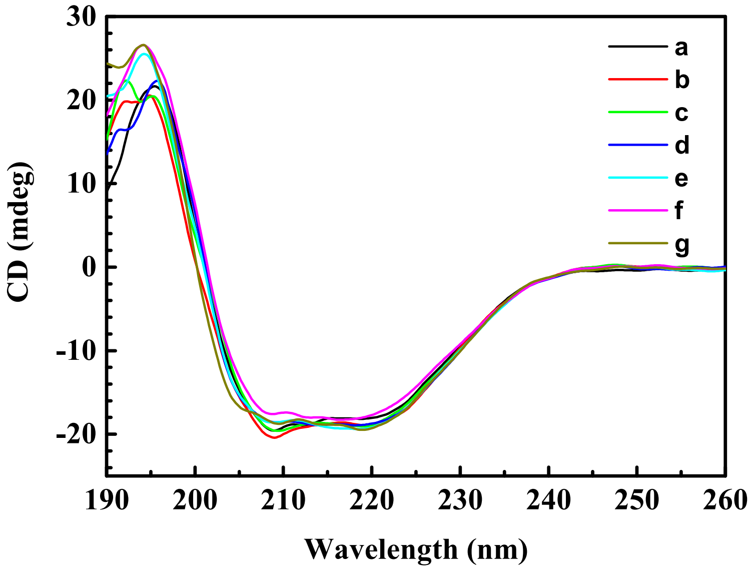

Fig. S4 CD spectra of CAT-ARS system.

Conditions: CAT: 1μM; ARS/(μM): a-g. 0, 2; 4, 6, 8, 10, 50; pH 7.4; T = 298 K.

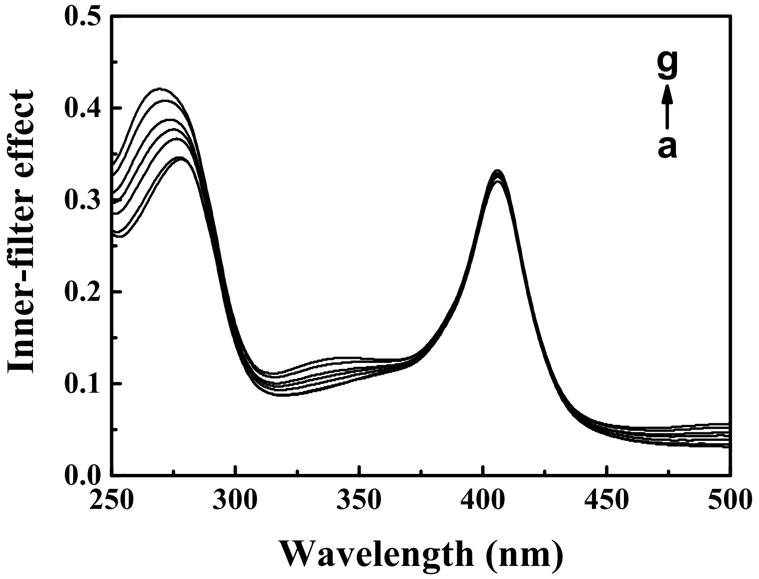

Fig. S5 The inner-filter effect of CAT-ARS system.

Conditions: CAT: 1μM; ARS/(μM): a-g. 0, 1, 2, 3, 4, 5, 6; pH 7.4; T = 298 K.

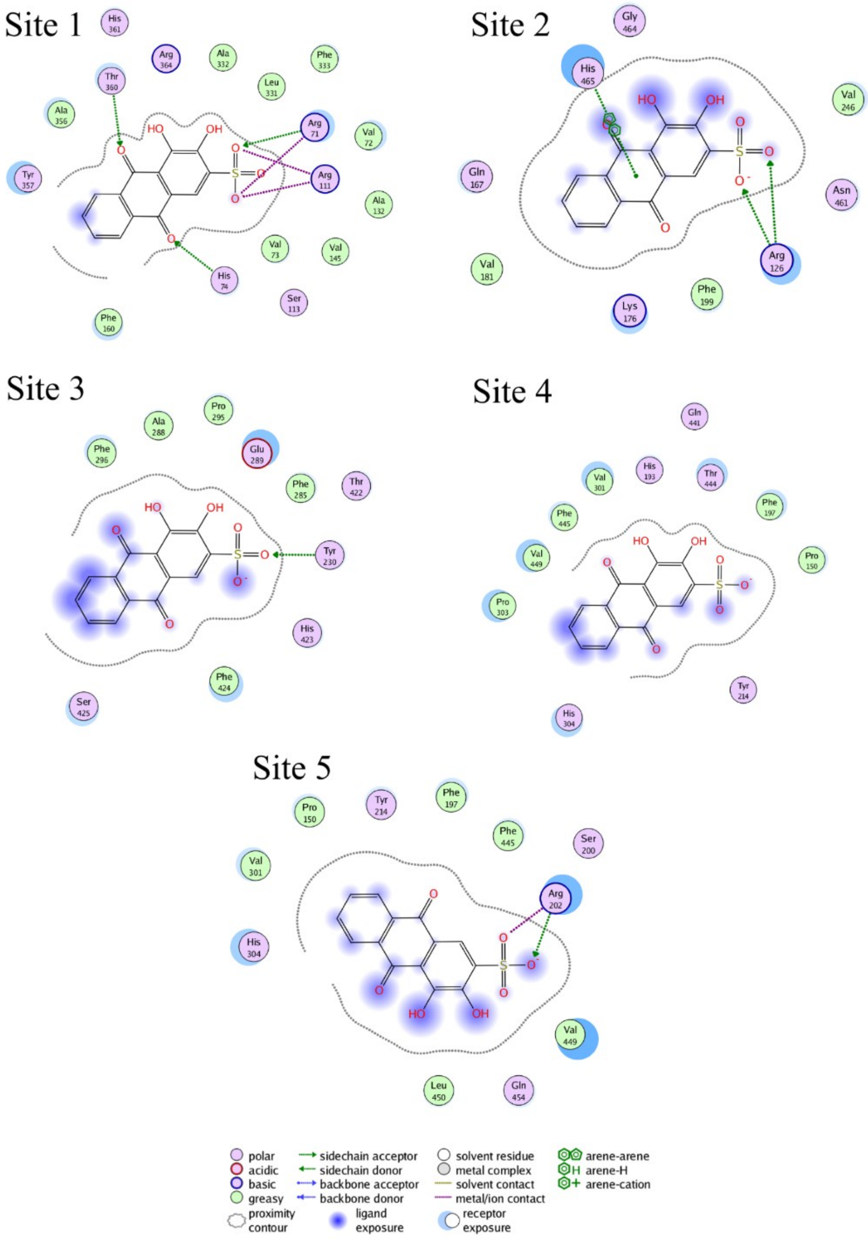

Fig. S6 Interaction between ARS and the amino acid residues in different binding sites of CAT.

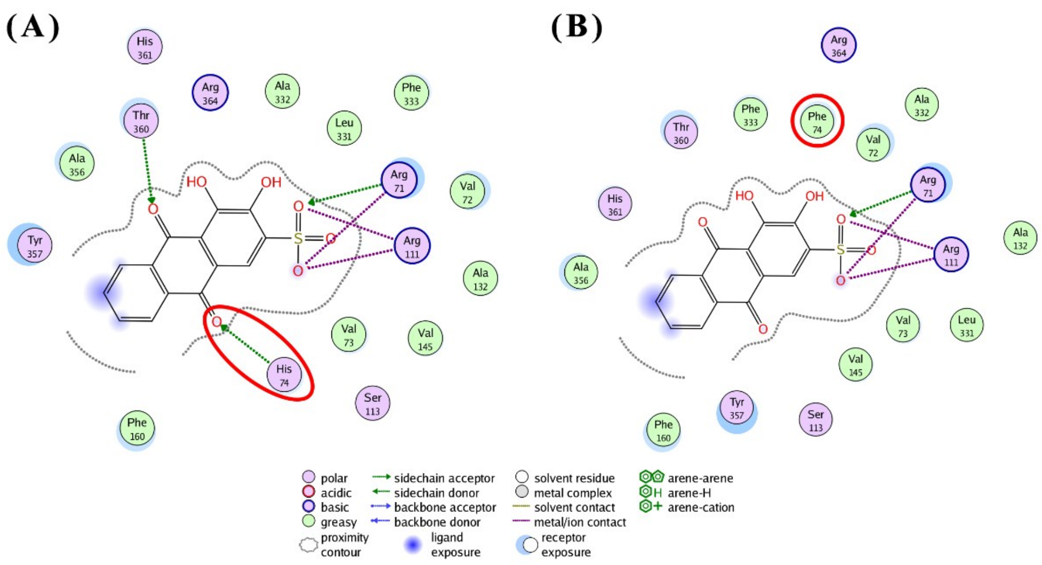

Fig. S7 Interaction between ARS and CAT: (A) original CAT; (B) CAT where His 74 was mutated to Phe.

Table S1 Binding parameters before and after mutating His74 at site 1.

Ligand	Receptor	Interaction	Distance	E (kcal/mol)
		original CAT		
O 8	CD2 HIS 74	H-acceptor	3.31	-0.7
O 11	OG1 THR 360	H-acceptor	3.42	-0.6
O 20	NE ARG 71	H-acceptor	2.83	-3.5
O 20	NE ARG 71	ionic	2.83	-5.7
O 20	NH2 ARG 71	ionic	3.67	-1.3
O 20	NE ARG 111	ionic	3.89	-0.7
O 21	NE ARG 71	ionic	3.8	-1
O 21	NE ARG 111	ionic	3.82	-0.9
		mutated CAT		
O 26	NE ARG 71	H-acceptor	2.82	-6
O 26	NE ARG 71	ionic	2.82	-5.8
O 26	NH2 ARG 71	ionic	3.66	-1.3
O 26	NE ARG 111	ionic	3.98	-0.6
O 27	NE ARG 71	ionic	3.73	-1.1
O 27	NE ARG 111	ionic	3.84	-0.8
